# Multidimensional Liquid Chromatography Employing a Graphene Oxide Capillary Column as the First Dimension: Determination of Antidepressant and Antiepileptic Drugs in Urine

**DOI:** 10.3390/molecules25051092

**Published:** 2020-02-29

**Authors:** Edvaldo Vasconcelos Soares Maciel, Ana Lúcia de Toffoli, Jussara da Silva Alves, Fernando Mauro Lanças

**Affiliations:** Institute of Chemistry of São Carlos, University of São Paulo, São Carlos, CEP 13566590, SP, Brazil; daltoniqsc@gmail.com (E.V.S.M.); ana_scalon@hotmail.com (A.L.d.T.); jussaraalves@usp.br (J.d.S.A.)

**Keywords:** liquid chromatography, mass spectrometry, sample preparation, automation, on-line, multidimensional, extraction column, urine, antidepressants, pharmaceutical drugs

## Abstract

Human mental disorders can be currently classified as one of the most relevant health topics. Including in this are depression and anxiety, which can affect us at any stage of life, causing economic and social problems. The treatments involve cognitive psychotherapy, and mainly the oral intake of pharmaceutical antidepressants. Therefore, the development of analytical methods for monitoring the levels of these drugs in biological fluids is critical. Considering the current demand for sensitive and automated analytical methods, the coupling between liquid chromatography and mass spectrometry, combined with suitable sample preparation, becomes a useful way to improve the analytical results even more. Herein we present an automated multidimensional method based on high-performance liquid chromatography-tandem mass spectrometry using a lab-made, graphene-based capillary extraction column connected to a C8 analytical column to determined five pharmaceutical drugs in urine. A method enhancement was performed by considering the chromatographic separation and the variables of the loading phase, loading time, loading flow, and injection volume. Under optimized conditions, the study reports good linearity with R^2^ > 0.98, and limits of detection in the range of 0.5–20 µg L^−1^. Afterward, the method was applied to the direct analysis of ten untreated urine samples, reporting traces of citalopram in one of them. The results suggest that the proposed approach could be a promising alternative that provides direct and fully automated analysis of pharmaceutical drugs in complex biological matrices.

## 1. Introduction

Diseases associated with human mental disorders can be currently classified as one of the most emergent topics in medicine. In this context are the widely known psychiatric illnesses called depression and anxiety. According to the World Health Organization, it is estimated that roughly 4.4% of the world population has already suffered from them. It is predicted that depression will be the second-most prevalent human disorder by 2030 [1].

In general, depression is considered a chronic disease that can arise in any stage of life, causing significant damage, including economic and social problems, and even leads to suicidal thoughts [2]. The most frequent symptoms of depression include unstable moods, fatigue, sadness, and insomnia. Additionally, anxiety can be considered another common type of psychiatric disorder that, when overlooked, leads to depression. In this case, arrhythmia, hyperventilation, sweating, racing thoughts, and insomnia indicate anxiety. Taking into account the similarities, there is presumably a direct correlation in terms of medical interventions. The most popular treatments involve cognitive psychotherapy, and mainly the use of pharmaceutical antidepressants (ADs) [3]. Therefore, considering the present panorama of mental disorders frequently reported in the 21st century, it is also expected that there will be an increase in antidepressant uptake by people in future.

Typically, these pharmaceutical drugs are divided into four main classes: tricyclic antidepressants (TCAs), selective serotonin reuptake inhibitor (SSRI), selective noradrenaline reuptake inhibitor (SNRI), and monoamine oxidase inhibitors (MOI) [4]. Although there are several different medicines commercially available, most of them have similar side effects (mainly in the early stages of administration), and a slow time to start acting on the human brain [5]. Besides these, other medications, such as antiepileptic drugs, can also be used to treat such disorders since they can act as mood stabilizers in some cases [6].

For these reasons, precise monitoring regarding their levels in the biological fluids is mandatory to guarantee therapeutic effectiveness and to diminish side effects. Moreover, the use of these drugs combined with other prescription medications may cause toxic problems, and, in the last few decades, their use for recreational purposes has concerned health organizations around the world [7,8]. Therefore, the development of analytical methods to determine the residues of ADs in human samples is very important in areas such as medicine and forensics. Several analytical techniques can be employed for these purposes, such as gas and liquid chromatography, capillary electrophoresis, and spectrophotometry, among others [1,9,10,11]. Considering the current demand for methods to be more sensitive and selective, the coupling between liquid chromatography and mass spectrometry becomes a useful way to improve the analytical results even more. Nonetheless, given the lower concentration levels of ADs and the complexity of biological samples, high-performance liquid chromatography-tandem mass spectrometry (HPLC-MS/MS) is not enough to achieve such results; hence, a previous step called sample preparation is often required [12].

Generally, these procedures are focused on removing interferents from the matrix, and on extracting/pre-concentrating target analytes [13]. The most common sample preparation techniques are conventional solid-phase extraction (SPE) and liquid–liquid extraction (LLE), which were proposed more than 50 years ago. These traditional approaches have many disadvantages, including laborious and time-consuming steps, large amounts of sample and solvent requirements, and disposable hardware (especially SPE), among other restrictions [14]. In order to overcome these shortcomings, modern sample preparation techniques based on the principles of the precursor solid-phase microextraction (SPME) began to appear in the early 1990s [14]. Consequently, the current trends are mainly based on miniaturization, automation, and high-throughput analysis, which point out automated methods that integrate sample preparation and HPLC-MS/MS as a suitable combination [15].

In this context, herein we propose an automated multidimensional method employing two columns, where the first one is specifically used for sample preparation and the second performs the chromatographic separation followed by tandem mass spectrometry detection. It is noteworthy that our capillary extraction column was packed with a lab-made extractive phase consisting of graphene oxide supported on an aminopropyl silica surface (GO-Sil). This column is much cheaper than the commercially available ones and has a reported excellent performance and robustness [16]. Additionally, the capillary dimensions of the extraction column (200-mm length and 508-µm i.d.) allow for economies in quantities of solvent, sample, and extractive phase, which are under the principles of green chemistry, which is so important nowadays. Its excellent extractive performance is attributed mainly to the high surface area of the graphene oxide, together with the delocalized π-electron system, which suggests a good affinity with molecules containing aromatic rings like the pharmaceutical drugs herein analyzed. In this case, the π-π interaction is the main interaction mechanism responsible for selective extraction. Aiming to evaluate the system performance, we selected four antidepressant drugs (ADs) as chemical probes, namely carbamazepine, citalopram, clomipramine, and desipramine, and one anticonvulsant AC, namely sertraline.

## 2. Results and Discussion

### 2.1. Method Enhancement

#### 2.1.1. Chromatographic Separation

**Figure 1:**
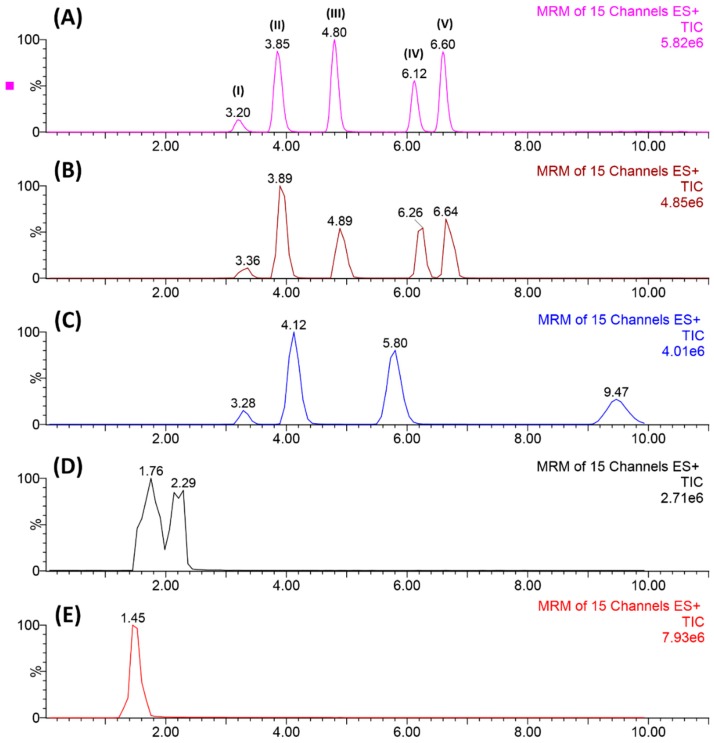
Representation of the chromatographic separation enhancement from E → A: (**A**) best condition applying elution gradient (H_2_O/ACN + 0.2% formic acid), (**B**) satisfactory separation but the dwell-time was not adjusted, (**C**–**E**) mobile phase without acidification and mobile flow rate not adjusted. Elution order: (I) carbamazepine, (II) citalopram, (III) desipramine, (IV) sertraline, and (V) clomipramine.

**Figure 2:**
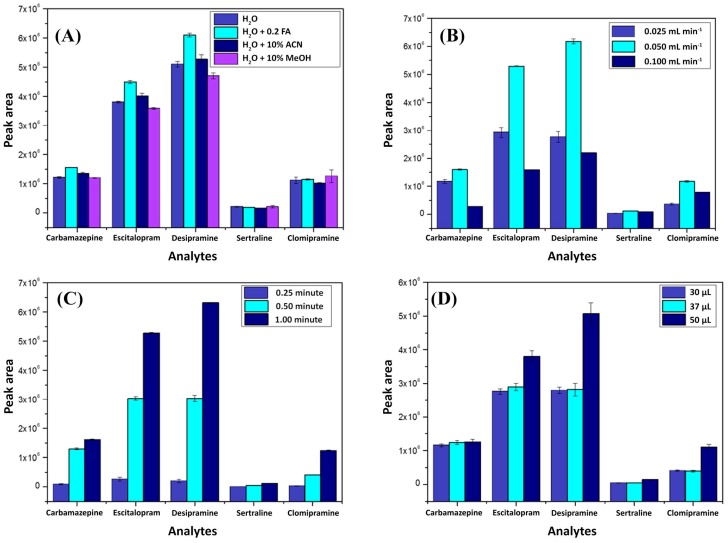
Method enhancement parameters obtained by univariate experiments considering the following parameters: (**A**) loading phase, (**B**) loading flow, (**C**) loading time, and (**D**) injection volume.

During the early stages of this work, experiments were performed that aimed to optimize the analytes’ chromatographic separation. Figure 1 illustrates the main results obtained by varying the mobile phase composition. As can be seen, our first attempt using isocratic mode (Figure 2E) reported a lower chromatographic resolution. However, as we were evaluating different combinations of mobile phases (D → B), improvements on the resolution were achieved. Finally, Figure 2A shows the best conditions regarding the separation of the five target analytes. In this case, an elution gradient employing ultrapure water and acetonitrile, both acidified with 0.2% formic acid, reported the best results. These gains in the resolution using the elution gradient might be due to the similarities in the analytes’ chemical structure, which required subtle variations on the mobile phase elution strength, in order to separate one from another compound. Additionally, as our mass spectrometer operated in electrospray (ESI) positive mode, which is known to suffer from a matrix effect that might lead to ion suppression or enhancement, the acidification of the mobile phases could aid the analytes to be more ionizable, increasing the analytical signal.

#### 2.1.2. Multidimensional Automated Procedure

In the sequence, a batch of experiments aiming to achieve an ideal analytical condition for all other influential parameters was conducted. Figure 2 depicts the results obtained for each investigated variable through univariate experiments by considering the area under the chromatographic peak as the response variable. All parameters were studied using triplicate injections. It is important to emphasize that when a parameter was not being evaluated, it was kept in the following standard analytical conditions: loading phase, H_2_O; loading flow, 0.05 mL min^−1^; loading time, 0.5 min; and injection volume, 50 µL.

First, the best composition of the loading mobile phase was evaluated. As can be seen in Figure 2A, the best extraction performance was reported using ultrapure water with formic acid (0.2%). This behavior can be explained due to the lower pH (≈3.2) obtained when formic acid (FA) is used, which can favor the interactions between the analytes and the sorbent phase. In this pH range, most molecules are charged and consequently have more affinity for the polar oxygen groups present on the graphene oxide surface [17,18]. Apart from that, using methanol and acetonitrile in the loading phase is expected to produce a higher elution strength, which makes the sorption of the analytes in the extraction column difficult; they pass directly through it, going to waste. Sequentially, the loading flow was investigated using univariate experiments with three different values: 0.025, 0.050, and 0.100 mL min^−1^. Figure 2B depicts the results using 0.050 mL min^−1^, reporting the best performance for the majority of the analytes. As can be seen, the intermediate value had the best performance when comparing it with 0.025 mL min^−1^. This fact can be explained by considering that the lower flow rate value might not be enough to ensure that all analytes had passed through the extraction column at the time the valve was switched to the elution position, causing analytes not to be sorbed into the extraction columns. Conversely, when considering 0.05 mL min^−1^, a higher flow hampered the analytes since they were desorbed due to a more diluted condition or due to the higher force that pushed them inside the extraction column, resulting in lower extraction performance.

After determining the best characteristic of the loading phase composition and flow rate, the other parameters were studied. Figure 2C shows that by increasing the loading time in which the analytes were pumped inside the extraction column, a better extraction performance was achieved. This effect is reasonable since a greater loading time implies more interaction between the analytes and the sorbent phase. Therefore, 1 min was fixed as the selected loading time. Furthermore, the volume of the sample injected into the system was varied to include these three values: 30, 37, and 50 µL. As can be expected, the larger sample volume (50 µL) resulted in better extraction performance since this is directly proportional to the number of analytes available to interact with the extraction column. For this reason, 50 µL was fixed as the injection volume.

### 2.2. Figures of Merit

The figures of merit herein evaluated were determined according to the International Conference on Harmonization (ICH) guidelines [19].

**Figure 3:**
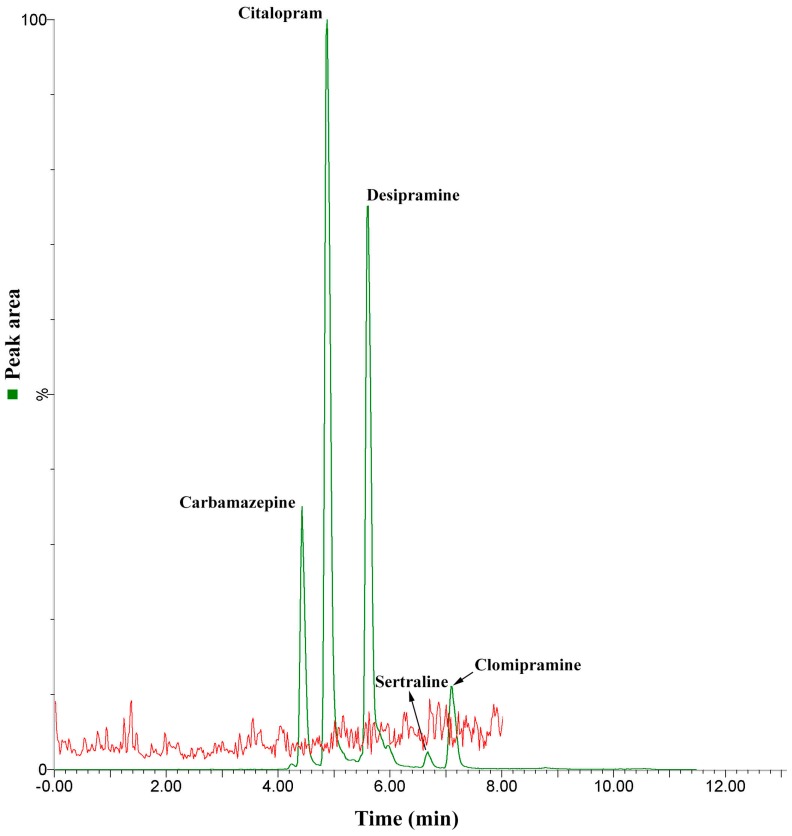
Chromatograms obtained by comparing a 100 µg L^−1^ spiked urine sample with an unspiked blank one in order to verify the selectivity of the proposed analytical method.

First, the method selectivity was evaluated by analyzing a sample obtained from a pool formed by blank urines, collected from consenting volunteers, which were compared with those obtained from the same sample after being spiked with a mixture containing the target analytes. As no peaks were observed in the multiple reaction monitoring (MRM) ion transition for each compound, the method was considered as being selective (Figure 3). In the sequence, the limits of detection and quantification were determined via successive injections of spiked urine samples until observing a signal to noise ratio near to 3:1 and 10:1, for LOD and LOQ, respectively. Therefore, the limits of detection ranged from 0.01–2.0 µg L^−1^ and the limits of quantification from 0.5–20 µg L^−1^. The method linearity was determined considering six different concentration levels, with each one being evaluated on triplicate injections. The linear interval for each analyte was: 1–200 µg L^−1^ for carbamazepine, citalopram, and desipramine, and 20–200 µg L^−1^ for sertraline and clomipramine. As shown in Table 1, the method presented good linearity with correlation coefficients (R^2^) higher than 0.985.

Afterward, the method accuracy, precision, and enrichment factor were all determined by considering three concentration levels (low, medium, and high) evaluated using injection triplicates. As can be seen in Table 2, the method presented good accuracy, with the values being between 83.2 and 117.6, which is considered acceptable according to the ICH guidelines (80–120%). Sequentially, the intra-day precision was determined on the same day of those other validation parameters, while the inter-day precision was evaluated on a subsequent day. Table 2 shows the obtained relative standard deviation (RSD) values, ranging from 1.4–13.6%, which were also per the ICH guidelines. Finally, as our analytical method was based on a multidimensional automated approach, it was essential to study the enrichment factor obtained by pushing the analytes through the extraction column before chromatographic analysis. In general, an increase in the analytical signal is expected when a pre-concentration step is carried out. Table 2 shows the obtained results for it, highlighting a good enrichment factor for all target compounds providing a signal enhancement varying from 4.7 to 59.4 when compared to the direct injection approach. Therefore, these results support the choice for a multidimensional and automated method to perform sample preparation and determination of pharmaceutical drugs in complex samples as urine. Furthermore, it must be underscored that the exceptional robustness of the in-house prepared extractive phase GO-Sil packed into the capillary extraction column was used for more than 250 urine injections without losing its original performance.

### 2.3. Overall Method Performance

When looking to compare our obtained results with other published papers in the literature, we can underscore some advantages, as well as limitations. First, as our paper presents the use of a synthesized graphene-based sorbent packed into a capillary extraction column, its robustness is noteworthy, as just described, given that it was applied to more than 250 injections. As examples, other recent works pinpoint their lab-made extractive hardware being re-used five and seventy times without losing its efficiency, respectively [20,21]. Likewise, our developed extraction column surpasses by far the commercially available SPE cartridges, which can be ideally used only once. Furthermore, considering our automated multidimensional approach using two columns, the system required only 50 µL of urine with reduced reagent consumption and consequent waste generation [4,22,23]. The lack of steps demanding operator intervention due to the automation can lead to remarkable gains in analysis time (≈8 min), while it also diminishes analytical errors resulting from sample handling [4,23]. Another great quality of it is the capacity to perform the analysis of antidepressants and antiepileptics in undiluted and unprecipitated urine. As highlighted by Cai et al. [24], several methods developed to analyze ADs in urine have been carried out by considering a dilution step due to the high complexity of the samples. Finally, the LODs and LOQs of the proposed approach are in a similar range with most published works; although some methods can be more sensitive, our results provide a suitable range for its main goal [25,26]. From the authors’ point of view, the major limitation of this proposed methodology is in its system configuration, since it demands an auxiliary pump and a switching valve, which might consist of a restriction for some laboratories.

### 2.4. Method Application

**Figure 4:**
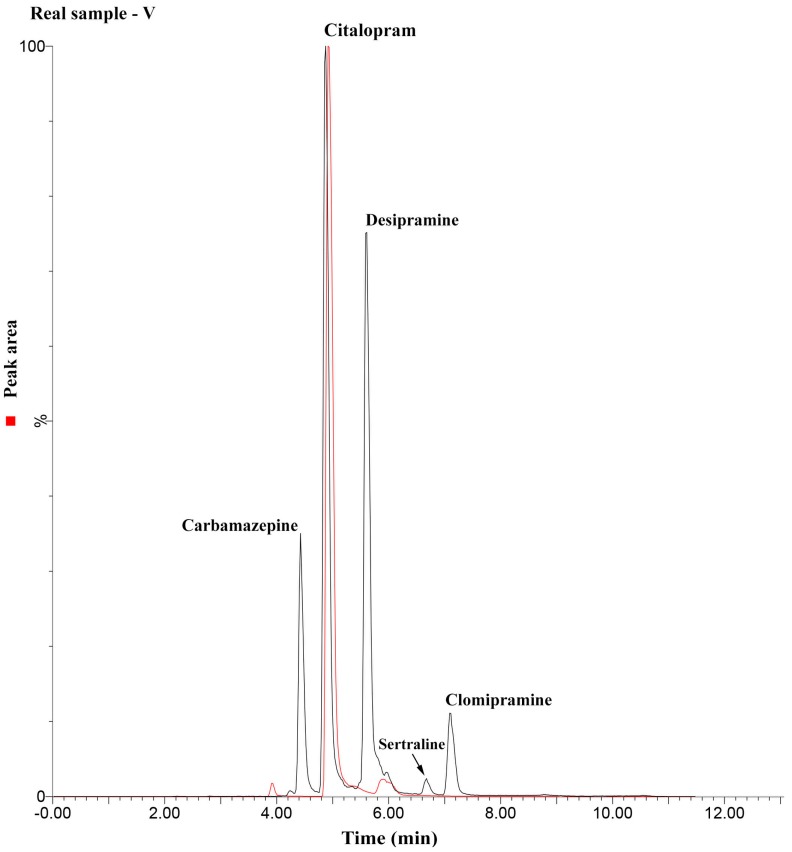
Chromatogram comparing a 150 µg L^−1^ spiked urine with a sample from a volunteer (red line) in which traces of citalopram were found.

Separately from the pool of blank samples used during the development step, the analytical methodology herein described was applied to the analysis of other urine samples collected from consenting volunteers. From ten samples analyzed for the target compounds, one presented traces of citalopram in a concentration estimated to be in the order of 150 µg L^−1^. This result is probably due to the considerably widespread use of citalopram (SSRI) at present since it has a broad spectrum of action, treating not only depression, but also obsessive-compulsive disorder, panic disorder, and social phobia [26]. Figure 4 shows the results comparing the referred sample (red line) with a blank one fortified with the analytes in a concentration range that resulted in an area similar to that obtained for the unknown sample. As can be seen, the signals for citalopram were in similar magnitude; the MRM transitions, the relative ratio between the monitored ions, and similarity of the retention time verifies the observed results.

## 3. Experimental

### 3.1. Reagents and Standard Solutions

High purity (99%) analytical standards of carbamazepine, citalopram, clomipramine, desipramine, and sertraline were all acquired from Fluka Analytical (St Louis, MO, USA). The analytes’ stock solutions were all prepared in methanol at a concentration of 1000 mg L^−1^, and subsequently diluted to 100 mg L^−1^. The work solutions were prepared from the stock ones in a proper concentration by considering the goal of each experiment to be performed. It should be highlighted that all standard solutions were temperature-controlled (−30 °C) inside the amber flasks.

The HPLC grade solvents acetonitrile (ACN) and methanol (MeOH) were purchased from TEDIA (Farfield, OH, USA) and the ultrapure water was produced at our laboratory using a MILLI-Q purification system from Millipore (Burlington, MA, USA). Furthermore, MS grade formic acid (FA) acquired from Sigma-Aldrich (St Louis, MO, USA) was used to acidify the chromatographic mobile phases. The GO-Sil extractive phase was synthesized and had already been used in previous works published by our research group [16,18].

### 3.2. Extraction Column Preparation

As our extraction column possessed capillary physical dimensions (200-mm length and 508-µm i.d.), our best choice to produce it was using the slurry packing procedure. In short, this consisted of using a high-pressure pump to push a suspension containing the stationary phase inside the column tubing, similar to that utilized in the production of HPLC and U-HPLC analytical columns. Therefore, the slurry packing system mainly consisted of a packing solvent, a slurry solvent to dissolve the stationary phase, a reservoir where the suspension was kept, and the column hardware often placed in the inferior part of the system.

In this work, a Haskell DSFH-300 hydropneumatic pump acquired from Haskel (Burbank, CA, USA) was employed as the pushing pump, while ultrapure water was used as the packing solvent. The suspension consisted of 10 mg of GO-Sil extractive phase dissolved in 700 µL of the slurry solvent (isopropanol/tetrahydrofuran; 6:1 *v*/*v*). The packing pressure was maintained at ≈600 bar during the procedure (≈60 min) in order to fill the column tubing. For more detailed information about the extraction column production, as well as for the GO-Sil extractive phase characterization assays (SEM and FTIR), please refer to a recent manuscript published by our research group [16].

### 3.3. Instrumentation

The analytical system was composed of an Acquity UPLC liquid chromatograph equipped with a binary solvent manager, and a sample manager coupled to a Xevo TQ S mass spectrometer using electrospray ionization, all from Waters (Milford, MA, USA). Moreover, a Shimadzu LC 10A*i* equipped with a degasser 10A from Shimadzu (Kyoto, JAP), and an electronically assisted switching valve from Supelco (St. Louis, MO, USA) were used to carry out the automated sample loading step, transferring the sample from its original vial to inside the first (extraction) column.

The chromatographic separations were achieved using a Poroshell 120 SB-C8 analytical column from Agilent (Santa Clara, CA, USA) (100 mm × 2.1 mm × 2.7 µm d_p_) at a temperature of 40 °C. The mobile phase consisted of ultrapure water and acetonitrile (both acidified with 0.2% formic acid) at a flow rate of 0.20 mL min^−1^, and the loading phase contained acidified ultrapure water (0.2% formic acid) at a flow rate of 0.05 mL min^−1^.

The mass spectrometry parameters were optimized via direct infusion of each analyte in standard solutions at a concentration of 0.5 mg mL^−1^, assisted by the IntelliStart optimization software (4.1) from Waters (USA). Under the optimized conditions, the detection method included a positive ESI, capillary voltage of 3.9 kV, source temperature of 150 °C, desolvation gas (N_2_) temperature of 650 °C and flow of 1000 L h^−1^, and collision gas (Ar) flow of 0.15 mL min^−1^. In order to enhance the method selectivity, the MS/MS configuration operation in the multiple reaction monitoring (MRM) was chosen to be used. All the analytes’ transitions used for identification/quantification, as well as its main detection parameters, can be found in Table 3.

### 3.4. Multidimensional Analytical Method

**Figure 5:**
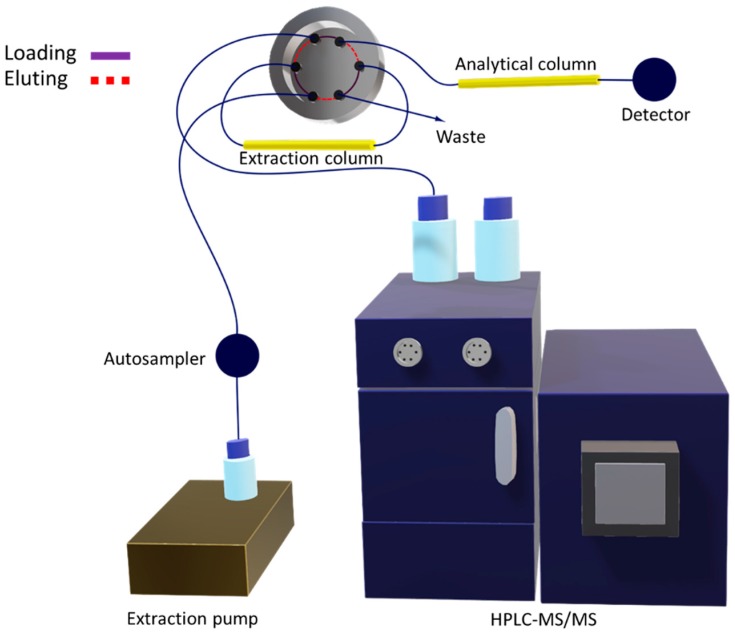
Illustrative drawing representing the multidimensional configuration, including the extraction column (first dimension) connected to the analytical column (second dimension) via a switching valve placed before the HPLC-MS/MS instrument.

The multidimensional analytical method was composed of two columns (extraction and analytical) connected using the switching valve, which was responsible for steering the flow depending on the purpose. Figure 5 illustrates the configuration assembled to perform the automated analysis.

Before starting any analysis, the urine samples were simply filtered through a 0.22-µm cellulose membrane to avoid clogging the whole system.

During each analysis, the autosampler was responsible for controlling the chromatographic injection and the valve positions. This was done through a sequence of events scheduled in the software. First, the sample injection was performed with the valve set at the loading position (valve ports connected through the purple line; see Figure 5). Therefore, the LC 10A*i* auxiliary pump carried the sample through the capillary extraction column, at a flow of 0.05 mL min^−1^, in order to retain the analytes while the majority of interferents went to waste. Meanwhile, the HPLC binary solvent pump conditioned the analytical column with the initial composition of the elution gradient. After 1 min, the valve was switched to the eluting position (valve ports connected through the red dotted lines; see Figure 5). Thus, the chromatographic mobile phase was pumped inside the extraction column, at a flow rate of 0.2 mL min^−1^, to desorb the analytes, shifting them to the analytical column and further to the mass spectrometer. In the sequence, the multidimensional system was washed and conditioned again to be ready for the next injection. Table 4 summarizes the main steps regarding the described analytical procedure.

### 3.5. Method Enhancement

In order to achieve a satisfactory sample clean-up (eliminating the majority of endogenous urine compounds) combined with a good chromatographic resolution and MS detectability, a batch of univariate experiments were performed. Therefore, the influences of the elution gradient, injection volume, loading flow, loading time, and loading phase composition were all investigated. These experiments were performed via injection of triplicates of blank urine samples spiked at 100 µg L^−1^.

First, the chromatographic separation was studied by changing the mobile phase solvent composition as well as the pH. Three solvents were tested (MeOH, ACN, and H_2_O), and formic acid was added to modify the pH. Sequentially, four parameters directly related to the extraction column were considered: (i) the loading phase composition: H_2_O, H_2_O (0.2% FA), H_2_O/ACN, and H_2_O/MeOH; (ii) the loading flow: 0.025, 0.05, and 0.1 mL min^−1^; (iii) the loading time: 0.25, 0.5, and 1.0 min; and (iv) the injection volume: 30, 37, and 50 µL. The parameters and its evaluation conditions were chosen by considering our experience with such types of multidimensional configurations [16,25].

### 3.6. Figures of Merit

Afterward, a systematic study regarding the analytical figures of merit commonly considered for validation procedures was performed according to international guidelines [19]. Therefore, individual experiments were carried out by contemplating six different variables: linearity, accuracy, precision, limits of quantification and detection, pre-concentration factor, and selectivity. It is essential to highlight that the pool of urine samples used in this step was collected from consenting volunteers and previously tested to verify the absence of the analytes such that they could be considered blank samples that would not interfere with the spiked concentration levels.

The method linearity was studied through the matrix-matched calibration method by spiking urine samples at six different concentration levels: 1, 25, 50, 75, 100, 150, and 200 µg L^−1^ for carbamazepine, citalopram, and desipramine; 20, 40, 80, 100, 150, and 200 µg L^−1^ for sertraline; and 25, 50, 75, 100, 150, and 200 µg L^−1^ for clomipramine. Each concentration level was evaluated using triplicate extractions with the automated multidimensional approach. The limits of detection (LODs) and quantification (LOQs) were determined via comparison of the signal to noise ratio in blank samples and those spiked at known concentration levels. Determination of the LOD was chosen at a signal to noise ratio of 3:1, while for LOQ, a signal to noise ratio of 10:1 was considered. The selectivity was investigated via comparing the pool of “blank” urine with those spiked at known concentration levels to verify the absence of interferent signal on the compounds’ retention time or MRM transitions. First, the accuracy was determined in three different concentrations via measuring the actual value obtained from the linearity equation (C_r_) and comparing it with the theoretical concentration value of each spiking level on the analytical curve (C_t_). Sequentially, precision was studied in terms of the relative standard deviation (RSD %) at three different levels of concentration, repeated in two consecutive days (intra- and inter-day assays). Finally, the pre-concentration factor (or enrichment factor) was evaluated by performing several injections of spiked urine samples via employing the multidimensional system (passing through the extraction column), which were compared with those similarly spiked and were directly injected into the analytical column.

### 3.7. Method Application

Urine samples used in this work were collected from consenting volunteers. Part of it was prior analyzed for the presence of the target drugs; in its absence, they formed a pool of samples used as “blank samples” during all stages of the study development. Additionally, the other samples not tested were used to verify the method’s applicability after the determination of the figures of merit. All aliquots were only filtered through 0.22 µm cellulose membrane prior injection into the automated multidimensional system.

## 4. Conclusions

Herein an online automated analytical method based on multidimensional liquid chromatography coupled to tandem mass spectrometry was developed to extract and determine four antidepressants and one antiepileptic drug in human urine. The approach was based on the interconnection between two columns being the first accountable to perform the analytes’ extraction (first dimension) while the second worked as a chromatographic analytical column (second dimension). Our capillary extraction column was packed with a synthesized graphene-based sorbent that exhibits excellent extraction performance and robustness being used for more than 250 injections. The method takes roughly 8 min and used 50 µL of undiluted and unprecipitated urine, demanding only a simple filtration step before injection into the multidimensional system. Besides, essential parameters were investigated to find out an ideal analytical condition allowing the determination of some validation figures of merit: linearity, accuracy, precision, selectivity, enrichment factor, LOD, and LOQ. Afterward, all ten urine samples collected from the consenting individuals in the study were analyzed to verify the proposed procedure. The presence of citalopram residues at a concentration level of around 150 µg L^−1^ was found in one of the ten analyzed samples. Therefore, based on the results obtained and reported in this manuscript, the proposed multidimensional analytical method was revealed to be a promising way to perform rapid and effective trace analysis of antidepressant and antiepileptic drugs in urine that easily adaptable to work with other biological complex matrices, such as saliva and plasma, among others.

## Figures and Tables

**Table 1:** Method linearity characteristics and its limits of detection (LOD) and quantification (LOQ).

Analytes	Linear Equation	R^2^	LOD (µg L^−1^)	LOQ (µg L^−1^)
Carbamazepine	y = 1681.7 + 2835.6x	0.999	0.01	0.5
Citalopram	y = −2542.9 + 6904.3x	0.997	0.04	0.5
Clomipramine	y = −14200.5 + 1167.5x	0.985	0.5	25
Desipramine	y = −1593.3 + 7048.4x	0.994	0.01	0.5
Sertraline	y = −1614.1 + 128.4x	0.985	2.0	20

**Table 2:** Additional figures of merit including the method enrichment factor, accuracy, and precision. RSD: Relative Standard Deviation.

Analytes	Enrichment Factor			Accuracy (%)			Precision (% RSD)					
							Intra-Day			Inter-Day		
	L	M	H	L	M	H	L	M	H	L	M	H
Carbamazepine	4.7	5.3	5.1	83.2	95.8	98.8	12.3	2.3	3.6	13.6	2.1	1.4
Citalopram	6.8	7.6	7.0	125.3	89.7	99.1	2.0	1.9	3.2	6.8	2.9	5.5
Clomipramine	17.3	18.1	17.4	98.7	117.6	102.4	5.2	6.1	4.0	9.2	3.2	4.8
Desipramine	18.2	16.4	15.0	105.8	114.8	102.3	6.9	3.5	11.8	11.2	4.5	2.4
Sertraline	21.2	59.4	13.1	98.7	117.6	102.4	12.8	4.5	6.5	8.1	4.1	1.4

**Table 3:** Analytes’ multiple reaction monitoring (MRM) precursor and product ions and its main detection parameters.

Analyte	Precursor Ion (*m/z*)	Product Ion (*m/z*)	Cone Voltage (V)	Collision Energy (V)	Dwell Time (ms)
Carbamazepine	253	152	24	42	0.075
		167	24	44	0.075
		180	24	32	0.075
Desipramine	267	72	22	14	0.075
		193	22	42	0.075
		208	22	24	0.075
Sertraline	306	123	16	48	0.075
		159	16	30	0.075
		275	16	14	0.075
Clomipramine	315	58	24	30	0.075
		86	24	18	0.075
		227	24	42	0.075
Citalopram	325	109	32	30	0.075
		234	32	26	0.075
		262	32	20	0.075

**Table 4:** Analytical steps involved in the automated multidimensional extraction/determination of the analytes.

Event	Time (min)	Solvent Composition (Extraction Column)	Solvent Composition (Analytical Column)
Loading	0.00–1.00	H_2_O + 0.2% FA	H_2_O (A)/ACN (B) * (30%:70%)
Eluting	1.00–3.00	H_2_O (A)/ACN (B) * (30%:70% → 35%:65%)	H_2_O (A)/ACN (B) * (30%:70% → 35%:65%)
	3.00–6.00	H_2_O (A)/ACN (B) * (35%:65% → 40:60%)	H_2_O (A)/ACN (B) * (35%:65% → 40:60%)
	5.00–6.00	H_2_O (A)/ACN (B) * (40%:60%)	H_2_O (A)/ACN (B) * (40%:60%)
Cleaning	6.00–7.00	H_2_O (A)/ACN (B) * (40%:60% → 50%:50%)	H_2_O (A)/ACN (B) * (40%:60% → 50%:50%)
	7.00–7.66	H_2_O (A)/ACN (B) * (50%:50% → 10%:90%)	H_2_O (A)/ACN (B) * (50%:50% → 10%:90%)
	7.66–8.60	H_2_O + 0.2% FA	H_2_O (A)/ACN (B) * (10%:90%)
Conditioning	8.60–11.50	H_2_O + 0.2% FA	H_2_O (A)/ACN (B) * (30%:70%)

* Both mobile phases acidified with 0.2% formic acid.

## Abbreviations

ACN, acetonitrile; ADs, antidepressants; ESI, electrospray ionization; FA, formic acid; GO-Sil, graphene oxide supported onto aminosílica; HPLC, High-performance liquid chromatography; i.d., inner diameter; ICH, International Conference on Harmonization; LC, liquid chromatography; LLE, liquid–liquid extraction; LOD, limit of detection; LOQ, limit of quantification; MeOH, methanol; MRM, multiple reaction monitoring; MS, mass spectrometry; RSD, relative standard deviation; SPE, solid-phase extraction; SPME, solid-phase microextraction; SSRI, selective serotonin reuptake inhibitor; UPLC, ultra-performance liquid chromatography.
